# Supplementary material for: Insights into household transmission of SARS-CoV-2 from a population-based serological survey
Source: Nat Commun. 2021 Jun 15;12:3643. doi: 10.1038/s41467-021-23733-5 (PMC8206123; doi:10.1038/s41467-021-23733-5)
Supplement: Supplementary file 1 — Supplementary Information [file 41467_2021_23733_MOESM1_ESM.docx]

**Supplementary Information**

**Insights into Household Transmission of SARS-CoV-2 from a Population-based Serological Survey**

Qifang Bi^1^, Justin Lessler*^1^, Isabella Eckerle^2,3^, Stephen A Lauer^1^, Laurent Kaiser^2,4,5^, Nicolas Vuilleumier^5,6^, Derek AT Cummings^7,8^, Antoine Flahault^9,10,11^, Dusan Petrovic^12,13,14^, Idris Guessous^12,15^, Silvia Stringhini^12,13,15^, Andrew S. Azman*^1,11,12^

^1^Department of Epidemiology, Johns Hopkins Bloomberg School of Public Health

^2^Geneva Center for Emerging Viral Diseases and Laboratory of Virology, Geneva University Hospitals, Geneva, Switzerland

^3^Department of Microbiology and Molecular Medicine, Faculty of Medicine, University of Geneva, Geneva, Switzerland

^4^Division of Infectious Diseases, Geneva University Hospitals, Geneva, Switzerland

^5^Department of Medicine, Faculty of Medicine, University of Geneva, Geneva, Switzerland

^6^Division of Laboratory Medicine, Geneva University Hospitals, Geneva, Switzerland

^7^ Department of Biology, University of Florida, Gainesville, USA

^8^ Emerging Pathogens Institute, University of Florida, Gainesville, USA

^9^Division of Tropical and Humanitarian Medicine, Geneva University Hospitals, Geneva, Switzerland

^10^Department of Health and Community Medicine, Faculty of Medicine, University of Geneva, Geneva, Switzerland

^11^Institute of Global Health, Faculty of Medicine, University of Geneva, Geneva, Switzerland

^12^Division of Primary Care Medicine, Geneva University Hospitals, Geneva, Switzerland

^13^University Centre for General Medicine and Public Health, University of Lausanne, Lausanne, Switzerland

^14^Centre for Environment and Health, School of Public Health, Department of Epidemiology and Biostatistics, Imperial College London, London, UK

^15^Department of Health and Community Medicine, Faculty of Medicine, University of Geneva, Geneva, Switzerland

*Equal contribution

*Corresponding Author*

Andrew Azman

615 North Wolfe Street

Baltimore, Maryland, 21205

[azman@jhu.edu](mailto:azman@jhu.edu)

**
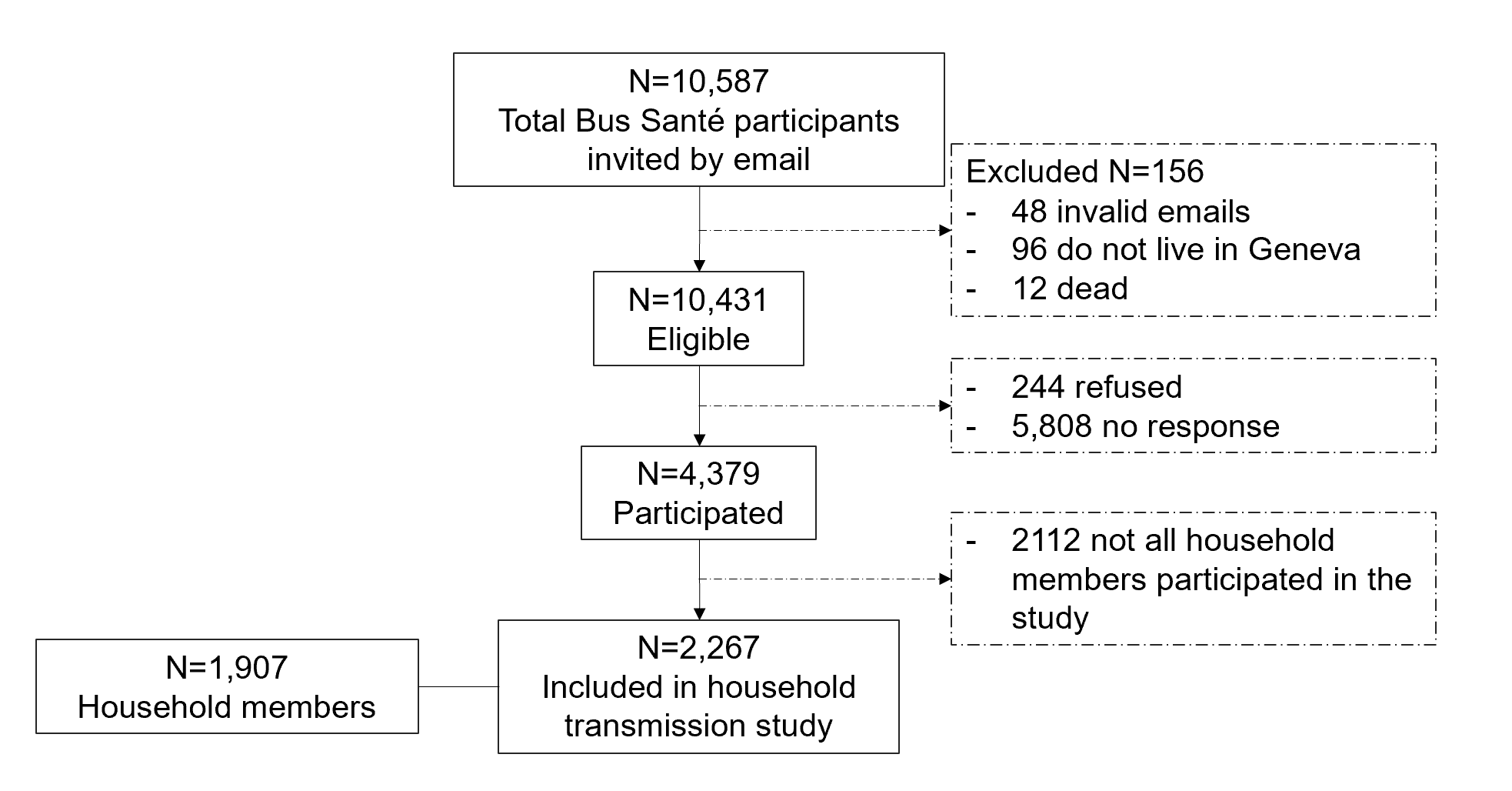
**

**Supplementary Figure 1.** Study enrollment flowchart. Comparison of the characteristics of those excluded due to being in an incomplete household to those included in the main analysis are shown in Supplementary Table 5.


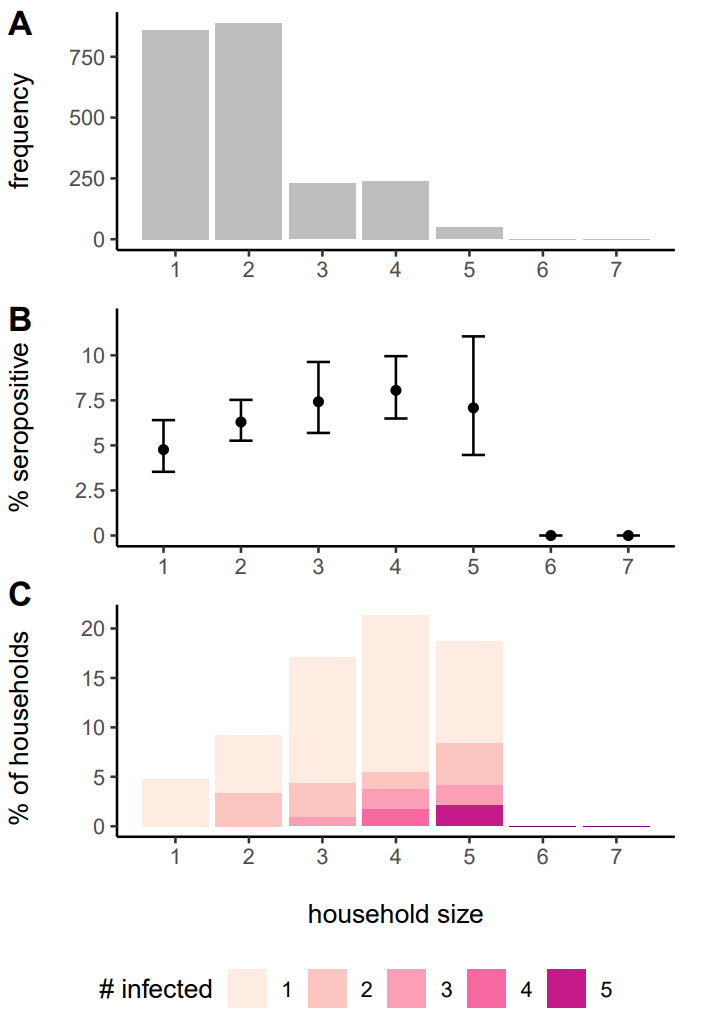


**Supplementary Figure 2**. Seropositivity by household size. A) Number of enrolled households by household size. 860, 889, 220, 239, 48, 1, and 1 households of size 1 to 7 were enrolled. B) Seropositive rate of individuals living in households of size 1 to 7. Seropositivity rates from left to right: 41/860, 112/1778, 51/687, 77/956, 17/240, 0/6, and 0/7. C) Distribution of number of seropositive people in a household by size. Summary statistics are presented in Supplementary Table 7.


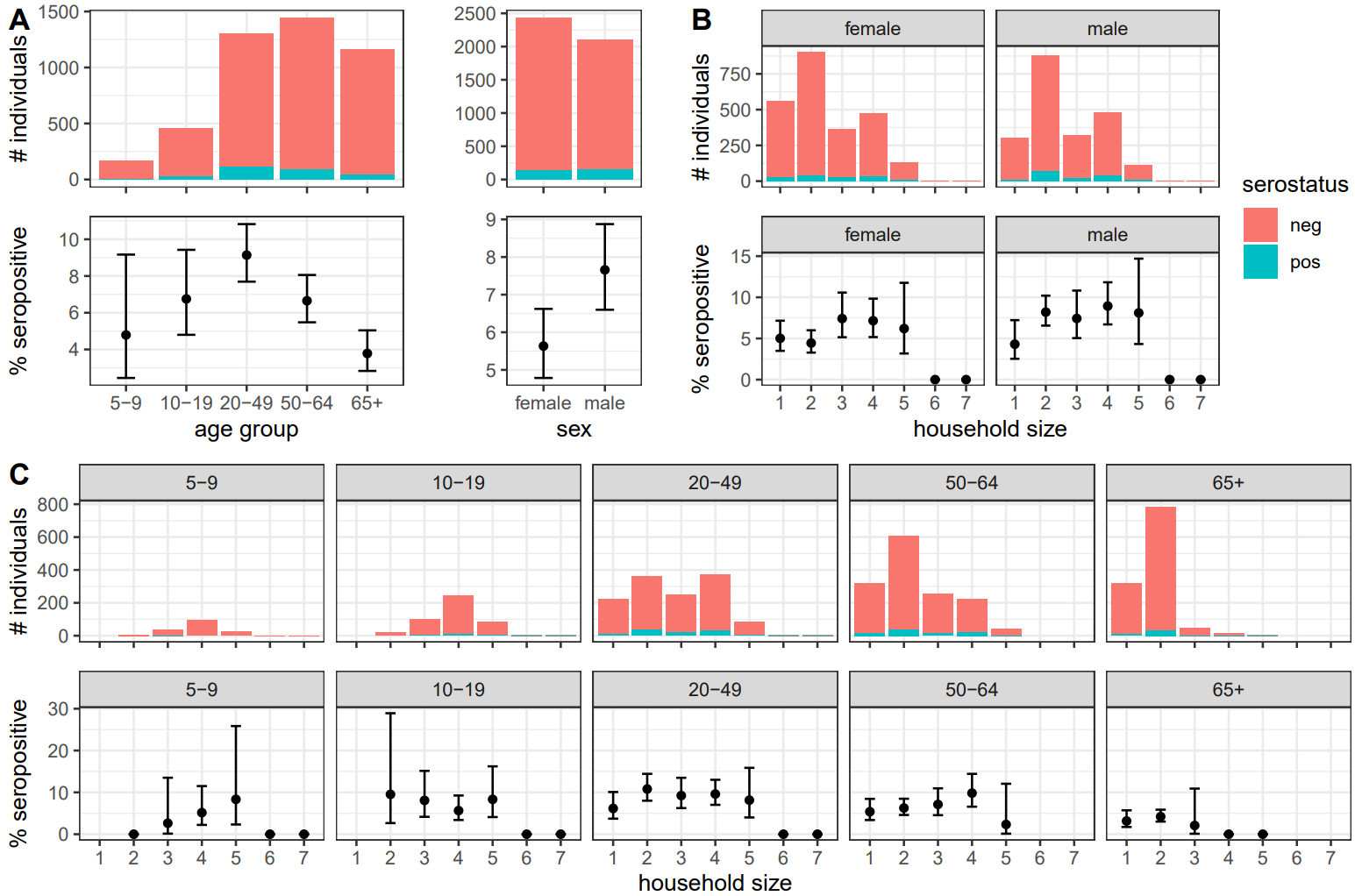


**Supplementary Figure 3**. Study population by age group, sex, and household size. A) Number of individuals and seropositivity rate by age group, and by sex. % seropositive from left to right: 8/167,31/459,119/1302,96/1443,44/1163;137/2432,161/2102. B) Number of individuals and seropositivity rate by household size and sex. % seropositive from left to right: 28/558,40/900,27/364,34/475,8/129,0/2,0/4; 13/302,72/878,24/323,43/481,9/111,0/4,0/3. C) Number of individuals and seropositivity rate by household size and age group. % seropositive from left to right: 0/0,0/6,1/38,5/97,2/24,0/1,0/1; 0/0,2/21,8/99,14/248,7/84,0/3,0/4; 14/227,39/361,23/249,36/375,7/86,0/2,0/2; 17/316,38/607,18/253,22/224,1/43,0/0,0/0; 10/317,33/783,1/48,0/12,0/3,0/0,0/0. Bar plots show the number of individuals in each group. Those tested seropositive and seronegative were colored in red and green respectively. The interval plots show seropositivity and accompanying 95% exact binomial confidence intervals in each group. Data that correspond to this figure are shown in Table 1.


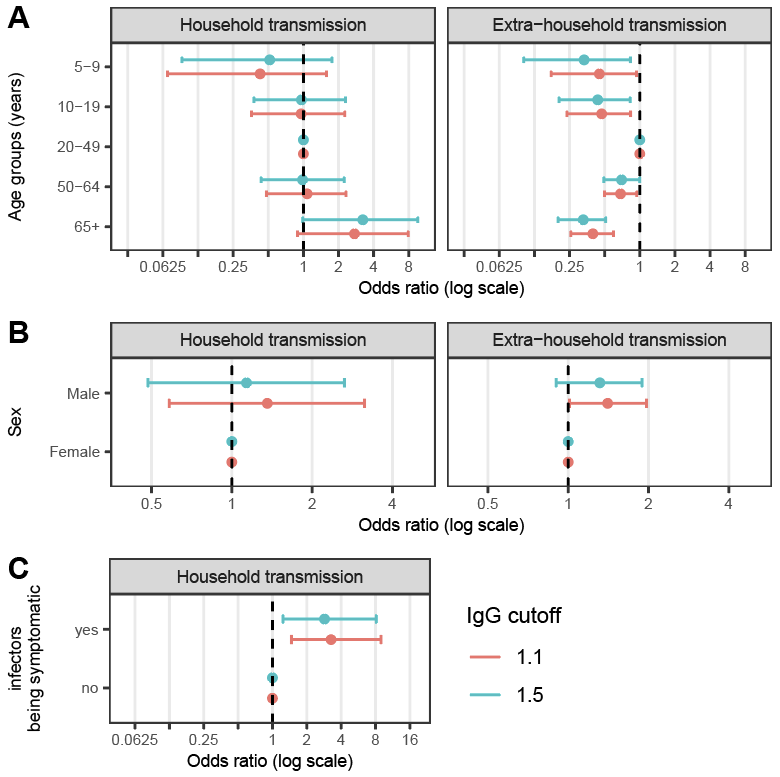


**Supplementary Figure 4**. Relative odds of being infected (i.e., tested seropositive) by individual characteristics using different definitions of seropositivity. In the main analysis, all samples with an optical density to cutoff ratio ≥1.1 were classified as being seropositive. In the sensitivity analysis, all samples with an optical density to cutoff >1.5 were classified as being seropositive. An odds ratio greater than 1 indicates infection is more likely to occur in this group compared to the reference group. The reference group for the age-specific and sex-specific odds ratio are 20-49 years old and female, respectively. The reference group for symptom status of potential infectors was infectors being asymptomatic. Dots and bars represent median and 95% credible intervals of the posterior distribution.


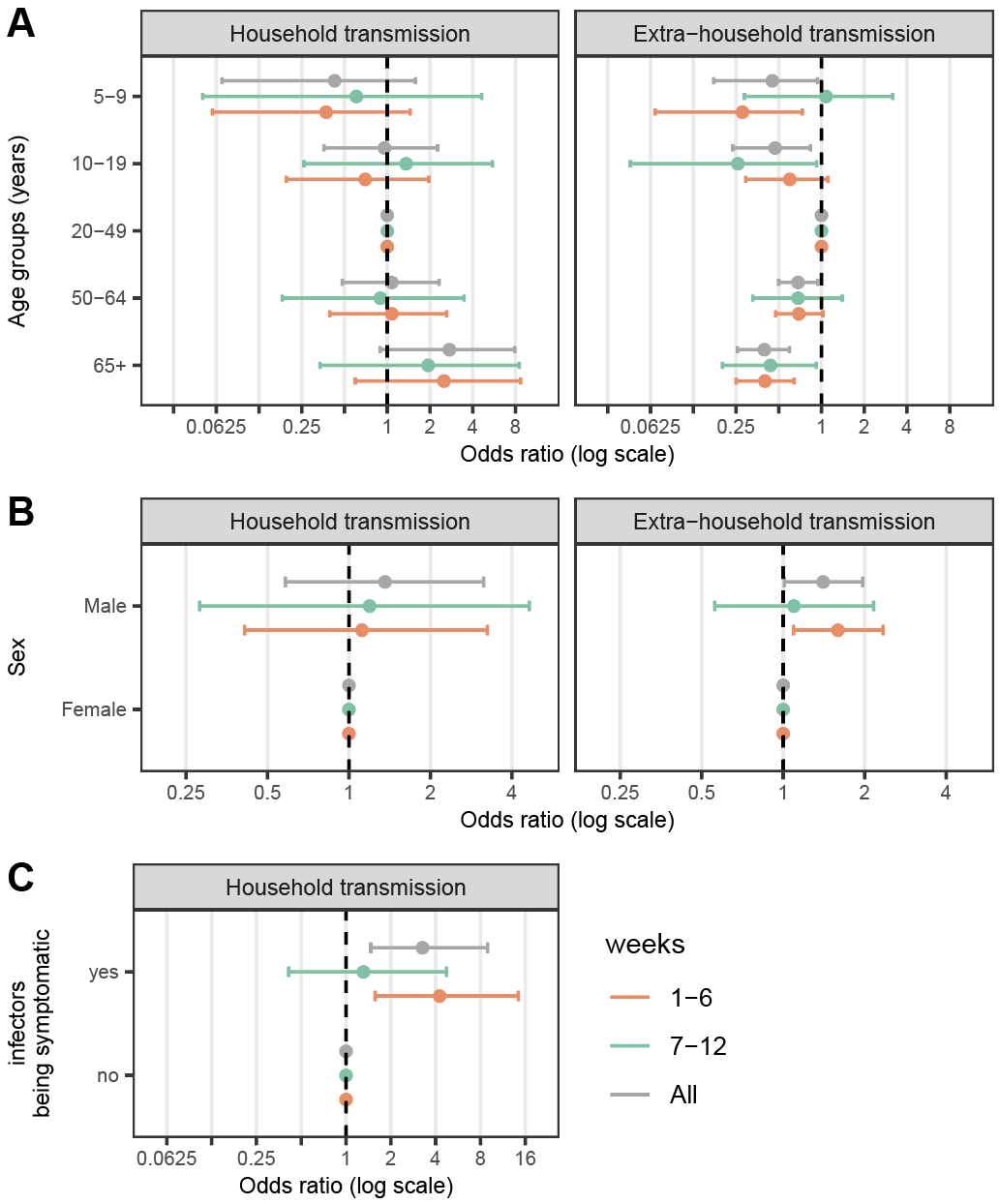


**Supplementary Figure 5.** Relative odds of being infected by individual characteristics in the first and second half of the study period**.** First half of the study period (first 6 weeks) spans from April 3rd to May 16th, and the second half (last 6 weeks) spans from May 18th to June 30th. An odds ratio greater than 1 indicates infection is more likely to occur in this group compared to the reference group. The reference group for the age-specific and sex-specific odds ratio are 20-49 years old and female, respectively. The reference group for symptom status of potential infectors was infectors being asymptomatic. Dots and bars represent median and 95% credible intervals of the posterior distribution.


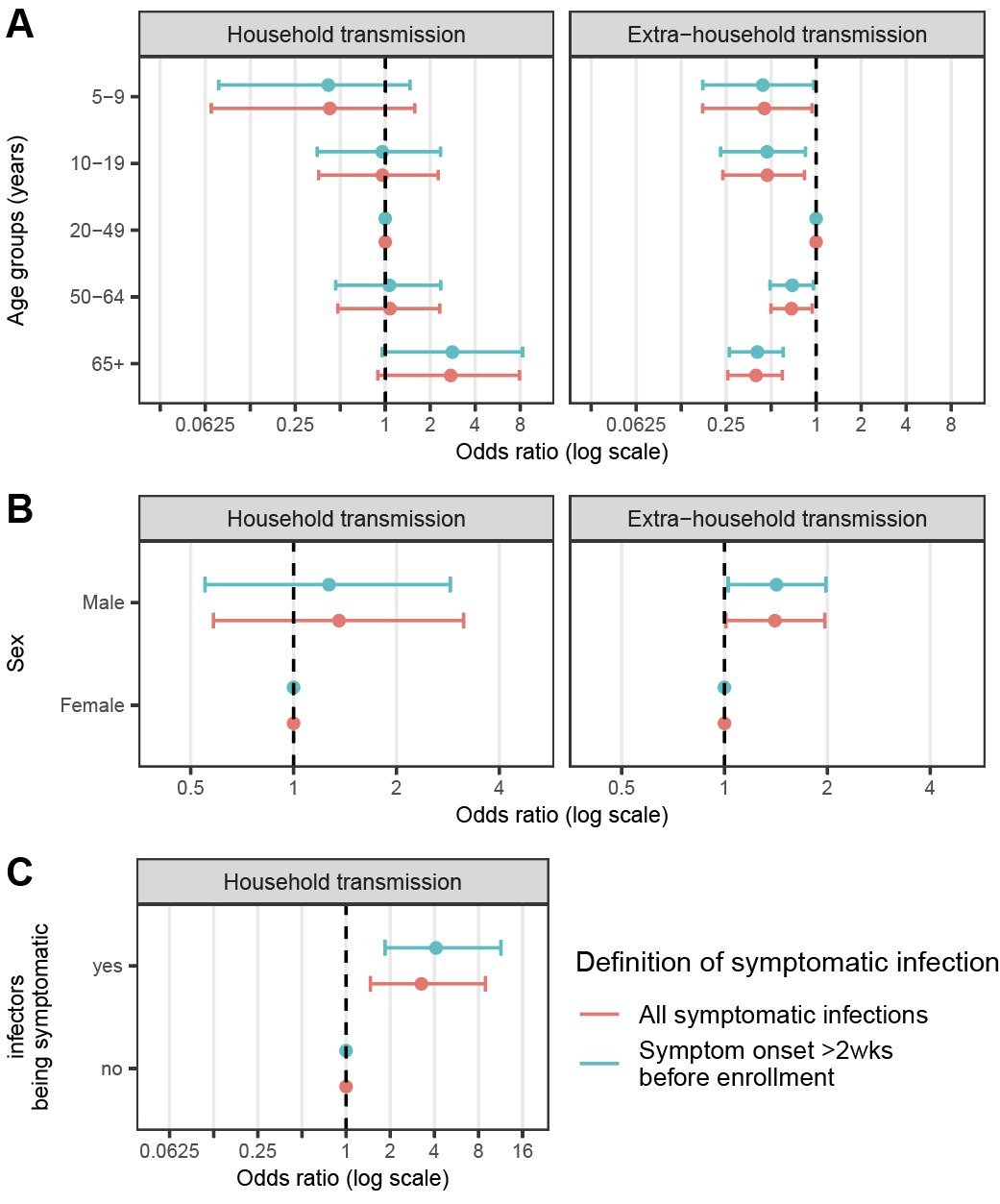


**Supplementary Figure 6**. Relative odds of being infected by individual characteristics by different definitions of symptomatic cases. For sensitivity analyses, only seropositive individuals reporting symptoms more than two weeks before enrollment were considered symptomatic. An odds ratio greater than 1 indicates infection is more likely to occur in this group compared to the reference group. The reference group for the age-specific and sex-specific odds ratio are 20-49 years old and female, respectively. The reference group for symptom status of potential infectors was infectors being asymptomatic. Dots and bars represent median and 95% credible intervals of the posterior distribution.


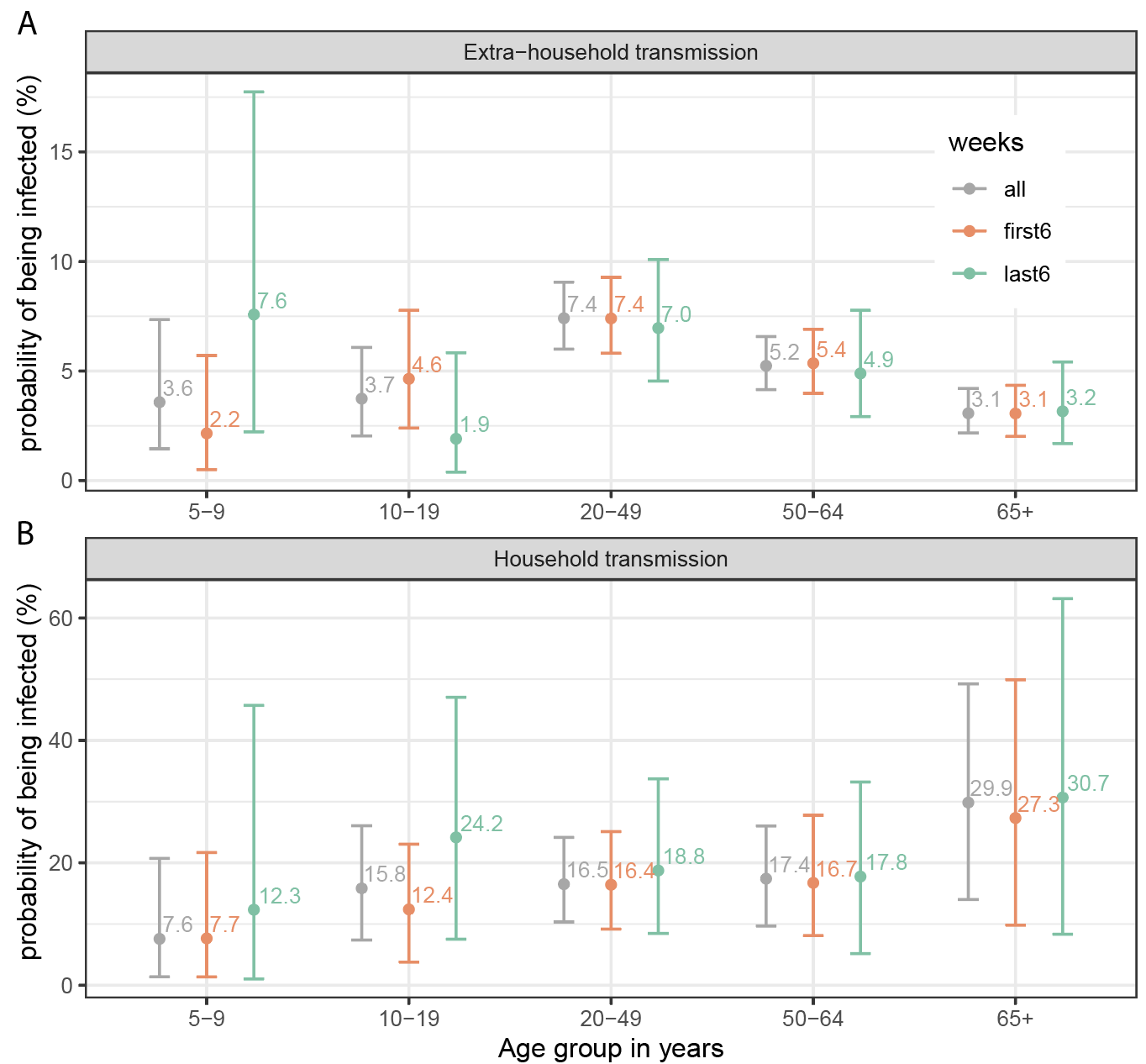


**Supplementary Figure 7**. Median probability of (A) extra-household infection over the duration of the outbreak and (B) infection from a single infected household member by age group and sex of the susceptibles in the first and second half of the study period. First half of the study period (first 6 weeks) spans from April 3rd to May 16th, and the second half (last 6 weeks) spans from May 18th to June 30th. Dots and bars represent median and 95% credible intervals of the posterior distribution.

**
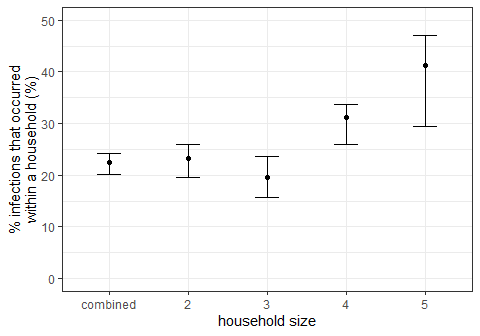
**

**Supplementary Figure 8**. Proportion of infections that occurred within a household of various household size. Dots and bars represent median and 95% credible intervals of the posterior distribution.

**
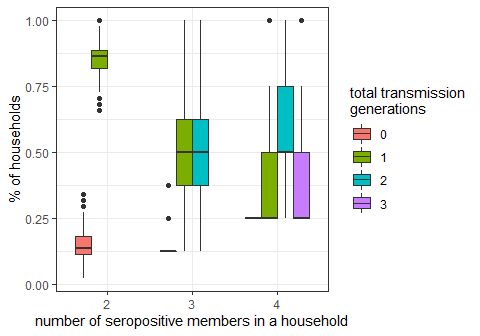
**

**Supplementary Figure 9.** Distribution of the number of transmission generations within households by number of seropositives in a household. Zero generations means that all members were infected outside of a household. One generation indicates that there is one generation of transmission within a household cause from one or more index cases. There are 44 households with two seropositive households members (8 households with 3 seropositives and 4 households with 4 seropositives). Dots and bars represent median and 95% credible intervals of the posterior distribution.

**Supplementary Table 1.** Household composition over the study period. HH stands for household. IQR stands for interquartile range.

| week | total households | % HHs with <10 years old (n) | % HHs with >65yrs old (n) | % HHs size = 1 (n) | average HH size (IQR) |
| --- | --- | --- | --- | --- | --- |
| 1 | 85 | 11.8% (10) | 18.8% (16) | 30.6% (26) | 2.33 (1, 3) |
| 2 | 111 | 5.4% (6) | 27.9% (31) | 29.7% (33) | 2.19 (1, 3) |
| 3 | 155 | 3.2% (5) | 32.9% (51) | 41.9% (65) | 1.95 (1, 2) |
| 4 | 153 | 9.8% (15) | 28.1% (43) | 33.3% (51) | 2.12 (1, 3) |
| 5 | 217 | 8.8% (19) | 25.8% (56) | 38.7% (84) | 2.07 (1, 3) |
| 6 | 226 | 4.9% (11) | 31.9% (72) | 35% (79) | 2.04 (1, 2) |
| 7 | 183 | 9.8% (18) | 31.1% (57) | 25.1% (46) | 2.33 (1.5, 3) |
| 8 | 218 | 2.3% (5) | 39.4% (86) | 45% (98) | 1.83 (1, 2) |
| 9 | 185 | 6.5% (12) | 40.5% (75) | 39.5% (73) | 1.98 (1, 2) |
| 10 | 232 | 4.7% (11) | 36.2% (84) | 37.9% (88) | 1.92 (1, 2) |
| 11 | 232 | 3.4% (8) | 47.4% (110) | 40.1% (93) | 1.81 (1, 2) |
| 12 | 270 | 4.4% (12) | 48.5% (131) | 45.9% (124) | 1.84 (1, 2) |
| Total | 2267 | 5.8% (132) | 35.8% (812) | 37.9% (860) | 2 (1, 2) |

**Supplementary Table 2.** Model performance and estimated parameters of the adapted chain binomial models that incorporate key individual-level factors (e.g., age, sex, reduced extra-household exposure, extra-household contact frequency of the exposed individuals and symptom status of the potential infectors) that may be associated with risk of infection from extra-household sources and by a single infected household member. Odds ratio over 1 indicates higher risk of infection. CrI stands for credible interval.

|  | Model 1 | | Model 2 | | Model 3 | |
| --- | --- | --- | --- | --- | --- | --- |
|  | age | | age + sex | | age/sex interaction | |
|  | extra-household | household | extra-household | household | extra-household | household |
| Category⤉ | Odds Ratio (95% CrI) | Odds Ratio (95% CrI) | Odds Ratio (95% CrI) | Odds Ratio (95% CrI) | Odds Ratio (95% CrI) | Odds Ratio (95% CrI) |
| Age |  |  |  |  |  |  |
| 5-9 | 0.5 (0.2, 1.0) | 0.4 (0.1, 1.4) | 0.5 (0.2, 1.0) | 0.4 (0.1, 1.5) | 0.4 (0.1, 1.1) | 0.3 (0.0, 1.4) |
| 10-19 | 0.5 (0.2, 0.9) | 0.9 (0.3, 2.3) | 0.5 (0.2, 0.9) | 0.9 (0.3, 2.1) | 0.4 (0.1, 0.9) | 1.4 (0.4, 4.6) |
| 20-49 | ref | ref | ref | ref | ref | ref |
| 50-64 | 0.7 (0.5, 1.0) | 1.1 (0.5, 2.3) | 0.7 (0.5, 1.0) | 1.0 (0.4, 2.4) | 0.6 (0.4, 1.0) | 0.8 (0.2, 2.6) |
| 65+ | 0.4 (0.3, 0.6) | 2.2 (0.7, 5.6) | 0.4 (0.3, 0.6) | 2.2 (0.8, 5.9) | 0.3 (0.2, 0.6) | 1.1 (0.2, 4.3) |
| Sex |  |  |  |  |  |  |
| Male | - | - | 1.4 (1.0, 1.9) | 1.6 (0.6, 3.9) | 1.1 (0.7, 1.7) | 1.6 (0.5, 5.2) |
| Female | - | - | ref | ref | ref | ref |
| Age-sex interaction |  |  |  |  |  |  |
| 5-9 Male | - | - | - | - | 1.3 (0.3, 6.1) | 1.7 (0.2, 13.1) |
| 10-19 Male | - | - | - | - | 1.4 (0.5, 4.7) | 0.4 (0.1, 2.1) |
| 20-49 Male | - | - | - | - | ref | ref |
| 50-64 Male | - | - | - | - | 1.3 (0.7, 2.6) | 1.7 (0.2, 13.1) |
| 65+ Male | - | - | - | - | 1.5 (0.7, 3.5) | 5.2 (0.7, 43.7) |
| ΔWAIC* | -14.8 | | -20.2 | | -19.2 | |

* WAIC of the null model (model 0) not model B or Q as a function of individual level characteristics is 2006.9. Change in WAIC is calculated relative to WAIC of the null model. Data for fitting model 5 and 6 include one less household due to missing contact variables. When fitting models 4-6 to the same dataset, model 4 has the lowest WAIC (ΔWAIC=-30.2).

⤉ Individual characteristics refer to the characteristics of the susceptibles if not specified.

|  | Model 4** | | *Model 5* | | Model 6 | |
| --- | --- | --- | --- | --- | --- | --- |
|  | age + sex + infectors’ symptom | | age + sex + infectors’ symptom + reduced contact | | age + sex + infectors’ symptom + reduced contact + extra HH contacts | |
|  | extra-household | household | extra-household | household | extra-household | household |
| Category⤉ | Odds Ratio (95% CrI) | Odds Ratio (95% CrI) | Odds Ratio (95% CrI) | Odds Ratio (95% CrI) | Odds Ratio (95% CrI) | Odds Ratio (95% CrI) |
| Age |  |  |  |  |  |  |
| 5-9 | 0.5 (0.2, 0.9) | 0.4 (0.1, 1.6) | 0.3 (0.1, 0.8) | 0.4 (0.1, 1.4) | 0.4 (0.1, 1.3) | 0.4 (0.1, 1.4) |
| 10-19 | 0.5 (0.2, 0.8) | 1.0 (0.4, 2.3) | 0.3 (0.2, 0.7) | 0.9 (0.3, 2.2) | 0.4 (0.1, 1.2) | 0.9 (0.3, 2.1) |
| 20-49 | ref | ref | ref | ref | ref | ref |
| 50-64 | 0.7 (0.5, 0.9) | 1.1 (0.5, 2.3) | 0.7 (0.5, 1.0) | 1.1 (0.5, 2.3) | 0.7 (0.5, 1.0) | 1.0 (0.4, 2.2) |
| 65+ | 0.4 (0.3, 0.6) | 2.7 (0.9, 7.9) | 0.2 (0.1, 0.9) | 2.7 (0.9, 7.4) | 0.3 (0.0, 1.5) | 2.8 (1.0, 7.5) |
| Sex |  |  |  |  |  |  |
| Male | 1.4 (1.0, 2.0) | 1.4 (0.6, 3.1) | 1.4 (1.0, 1.9) | 1.4 (0.6, 3.2) | 1.4 (1.0, 1.9) | 1.3 (0.6, 3.0) |
| Female | ref | ref | ref | ref | ref | ref |
| Symptomatic (infector) |  |  |  |  |  |  |
| yes | - | 3.3 (1.5, 8.9) | - | 3.3 (1.5, 8.4) | - | 3.4 (1.5, 8.5) |
| no |  | ref |  | ref |  | ref |
| Reduced contact among 20-64 yo |  |  |  |  |  |  |
| yes | - | - | 0.7 (0.4, 1.2) | - | 0.7 (0.4, 1.2) | - |
| no |  |  | ref |  | ref |  |
| Reduced contact among 65+ yo |  |  |  |  |  |  |
| yes | - | - | 1.1 (0.4, 4.3) | - | 1.1 (0.3, 5.7) | - |
| no |  |  | ref |  | ref |  |
| Extra household contacts among 20-64 yo |  |  |  |  |  |  |
| 0 | - | - | - | - | 1.6 (0.9, 2.9) | - |
| 1 to 2 | - | - | - | - | 1.2 (0.7, 2.0) | - |
| 6 to 10 | - | - | - | - | 1.8 (1.1, 3.0) | - |
| over 10 | - | - | - | - | 1.2 (0.7, 1.9) | - |
| Extra household contacts among 65+ yo |  |  |  |  |  |  |
| 0 | - | - | - | - | 0.5 (0.1, 1.8) | - |
| 1 to 2 | - | - | - | - | 1.6 (0.7, 4.1) | - |
| 6 to 10 | - | - | - | - | 2.0 (0.8, 5.2) | - |
| over 10 | - | - | - | - | 0.4 (0.1, 1.7) | - |
| ΔWAIC* | -29.6 | | -29.4 | | -30.0 | |

** Main model of the study; OR estimates of model 4 are presented in Figure 3.

⤉ Individual characteristics refer to the characteristics of the susceptibles if not specified

|  | Model 7 | | Model 8 | | Model 9 | |
| --- | --- | --- | --- | --- | --- | --- |
|  | age + sex + infectors’ symptom + infectors’ age | | sex + symptom + infectors’ age | | age + sex + infectors’ age | |
|  | extra-household | household | extra-household | household | extra-household | household |
| Category⤉ | Odds Ratio  (95% CrI) | Odds Ratio (95% CrI) | Odds Ratio (95% CrI) | Odds Ratio (95% CrI) | Odds Ratio (95% CrI) | Odds Ratio (95% CrI) |
| Age of infectees |  |  |  |  |  |  |
| 5-9 | 0.4 (0.2, 1.0) | 0.4 (0.1, 1.5) | - | - | 0.5 (0.2, 1.0) | 0.5 (0.1, 1.6) |
| 10-19 | 0.6 (0.2, 1.1) | 0.7 (0.2, 2.2) | - | - | 0.5 (0.2, 1.1) | 0.7 (0.1, 2.3) |
| 20-49 | ref | ref | - | - | ref | ref |
| 50-64 | 0.7 (0.5, 1.0) | 1.0 (0.4, 2.5) | - | - | 0.7 (0.5, 1.0) | 0.9 (0.3, 2.2) |
| 65+ | 0.4 (0.3, 0.6) | 1.8 (0.4, 9.0) | - | - | 0.4 (0.3, 0.6) | 2.1 (0.4, 8.0) |
| Sex |  |  |  |  |  |  |
| Male | 1.4 (1.0, 2.0) | 1.3 (0.5, 3.2) | 1.5 (1.1, 2.1) | 1.0 (0.5, 2.4) | 1.4 (1.0, 1.9) | 1.6 (0.7, 4.0) |
| Female | ref | ref | ref | ref | ref | ref |
| Symptomatic (infector) |  |  |  |  |  |  |
| yes | - | 3.6 (1.5, 10.4) | - | 3.9 (1.7, 10.6) | - | - |
| no | - | ref |  | ref | - | - |
| Age of infectors |  |  |  |  |  |  |
| 5-9 | - | 0.7 (0.0, 4.7) | - | 0.7 (0.0, 4.7) | - | 0.5 (0.0, 2.8) |
| 10-19 | - | 1.9 (0.2, 6.8) | - | 1.9 (0.2, 6.8) | - | 1.9 (0.2, 6.8) |
| 20-49 | - | ref | - | ref | - | ref |
| 50-64 | - | 1.2 (0.4, 3.6) | - | 1.2 (0.4, 3.6) | - | 1.2 (0.4, 3.6) |
| 65+ | - | 1.9 (0.3, 8.7) | - | 1.9 (0.3, 8.7) | - | 1.9 (0.3, 8.7) |
| ΔWAIC* | -24.7 | | -15.1 | | -15.5 | |

⤉ Individual characteristics refer to the characteristics of the susceptibles if not specified

**Supplementary Table 3**. Attributable fraction of extra-household infections, within household infections by symptomatics, and within household infections by asymptomatics. CrI stands for credible interval.

|  | Model 0; posterior median (95% CrI) | Model 1; posterior median (95% CrI) | Model 2; posterior median (95% CrI) | Model 3; posterior median (95% CrI) | *Model 4**; posterior median (95% CrI)* |
| --- | --- | --- | --- | --- | --- |
| % of all infections from transmission between households members | 22.5 (20.1, 24.2) | 22.5 (20.1, 24.2) | 22.5 (20.1, 24.2) | 22.5 (20.1, 24.2) | 22.5 (20.1, 24.2) |
| % of all infections from transmission between household members by household size |  |  |  |  |  |
| 2 | 23.2 (19.6, 25.9) | 23.2 (19.6, 25.9) | 23.2 (19.6, 25.9) | 23.2 (18.8, 25.9) | 23.2 (19.6, 25.9) |
| 3 | 21.6 (15.7, 23.5) | 19.6 (13.7, 23.5) | 19.6 (15.7, 23.5) | 19.6 (15.7, 23.5) | 19.6 (13.7, 23.5) |
| 4 | 31.2 (26.0, 33.8) | 29.9 (26.0, 33.8) | 29.9 (25.6, 33.8) | 29.9 (26.0, 33.8) | 31.2 (26.0, 33.8) |
| 5 | 41.2 (29.4, 47.1) | 41.2 (29.4, 47.1) | 41.2 (29.4, 47.1) | 41.2 (29.4, 47.1) | 41.2 (29.4, 47.1) |
| % of household infections from asymptomatic individuals | - | - | - | - | 14.5 (7.2, 22.7) |

|  | Model 5; posterior median (95% CrI) | Model 6; posterior median (95% CrI) | Model 7; posterior median (95% CrI) | Model 8; posterior median (95% CrI) | Model 9; posterior median (95% CrI) |
| --- | --- | --- | --- | --- | --- |
| % of all household infections from transmission between household members | 22.5 (20.5, 24.5) | 22.5 (20.5, 24.2) | 22.1 (20.1, 24.2) | 22.5 (20.5, 24.2) | 22.5 (20.0, 24.2) |
| % of all household infections from transmission between household members by household size |  |  |  |  |  |
| 2 | 23.2 (19.6, 26.8) | 23.2 (19.6, 25.9) | 23.2 (19.6, 25.9) | 23.2 (19.6, 25.9) | 23.2 (19.6, 25.9) |
| 3 | 19.6 (15.7, 23.5) | 19.6 (13.7, 23.5) | 19.6 (13.7, 23.5) | 19.6 (13.7, 23.5) | 19.6 (13.7, 23.5) |
| 4 | 31.2 (26.0, 33.8) | 31.2 (26.0, 33.8) | 29.9 (26.0, 33.8) | 31.2 (26.9, 33.8) | 29.9 (25.6, 33.8) |
| 5 | 41.2 (29.4, 47.1) | 41.2 (29.4, 47.1) | 41.2 (29.4, 47.1) | 41.2 (29.4, 47.1) | 41.2 (29.4, 47.1) |
| % of household infections from asymptomatic individuals | 14.7 (6.3, 23.2) | 14.5 (7.7, 22.2) | 14.3 (6.4, 22.7) | 13.0 (6.2, 20.3) | - |

** Main model of the study

**Supplementary Table 4**. Estimated probability of infection (%) from extra-household exposures from the start of the epidemic in Geneva until the time of the serosurvey and a single infected household member by age group and sex of the exposed individuals. Graphic representation of the results is shown in Figure 2 in main text. CrI stands for credible interval.

|  | Cumulative probability of infection from extra-household sources (95%CrI) | | | Household transmission probability from a single infected household member (95%CrI) | | |
| --- | --- | --- | --- | --- | --- | --- |
| Age group | All | Male | Female | All | Male | Female |
| 5-9 | 3.6 (1.4, 7.4) | 4.1 (1.6, 8.5) | 3.1 (1.2, 6.2) | 7.6 (1.4, 20.7) | 9.4 (1.7, 26.6) | 6.1 (1.3, 18.1) |
| 10-19 | 3.7 (2.0, 6.1) | 4.4 (2.3, 7.4) | 3.2 (1.7, 5.7) | 15.8 (7.4, 26.1) | 18.0 (8.2, 30.9) | 12.4 (4.7, 23.6) |
| 20-49 | 7.4 (6.0, 9.1) | 8.5 (6.6, 10.7) | 6.4 (4.8, 8.2) | 16.5 (10.3, 24.2) | 20.1 (11.0, 31.6) | 13.9 (6.8, 23.2) |
| 50-64 | 5.2 (4.2, 6.6) | 6.1 (4.7, 8.0) | 4.6 (3.3, 6.1) | 17.4 (9.7, 26.0) | 20.7 (10.5, 33.7) | 14.3 (6.7, 24.6) |
| 65+ | 3.1 (2.2, 4.2) | 3.5 (2.4, 5.0) | 2.6 (1.8, 3.7) | 29.9 (14.0, 49.2) | 36.2 (14.1, 59.6) | 26.1 (11.3, 45.8) |
| All | 5.1 (4.5, 5.8) | - | - | 17.3 (13.7, 21.7) | - | - |

**Supplementary Table 5**. Characteristics of those excluded due to being in an incomplete household to those included in the main analysis. P-values were obtained from chi-square tests.

|  | Study participants in complete households,  % (n); N=4,534 | Study participants in incomplete household,  % (n); N=3,810 | p-value |
| --- | --- | --- | --- |
| Age |  |  |  |
| 5-9 | 3.7% (167) | 2.8% (106) | <0.0001 |
| 10-19 | 10.1% (459) | 9.0% (344) |  |
| 20-49 | 28.7% (1302) | 42.8% (1632) |  |
| 50-64 | 31.8% (1443) | 32.8% (1251) |  |
| 65+ | 25.7% (1163) | 12.5% (477) |  |
| Serostatus |  |  |  |
| seropositive | 6.6% (298) | 7.7% (292) | 0.058 |
| seronegative | 93.4% (4236) | 92.3% (3517) |  |
| Age specific seropositivity rate |  |  |  |
| 5-9 | 4.8% (8/167) | 0.94% (1/106) | 0.019 |
| 10-19 | 6.8% (31/459) | 9.9% (34/344) |  |
| 20-49 | 9.1% (119/1302) | 8.8% (144/1632) |  |
| 50-64 | 6.7% (96/1443) | 6.7% (84/1251) |  |
| 65+ | 3.8% (44/1163) | 6.1% (29/477) |  |
| Sex |  |  |  |
| Female | 53.6% (2432) | 53.4% (2033) | 0.82 |
| Male | 46.4% (2102) | 46.6% (1777) |  |
| Self-reported symptoms among the seropositives |  |  |  |
| Symptomatic | 71.5% (213/298) | 75% (219/292) | <0.0001 |
| Asymptomatic | 28.5% (85/298) | 25% (73/292) |  |
| Education Level⤉ |  |  |  |
| Compulsory School | 8.5% (385) | 9.4% (357) | 0.051 |
| High School | 16.6% (751) | 17.2% (656) |  |
| Vocational School | 14.0% (637) | 12.6% (481) |  |
| College | 42.2% (1913) | 45.4% (1730) |  |
| Doctorate | 5.1% (229) | 4.6% (176) |  |
| Other | 4.6% (207) | 4.1% (155) |  |
| Missing Response | 9.1% (412) | 6.7% (255) |  |
| Reduced Contact |  |  |  |
| No | 4.9% (224) | 6.0% (229) | 0.055 |
| Yes | 86.3% (3914) | 86.8% (3308) |  |
| Missing Response | 8.7% (396) | 7.2% (273) |  |
| Employment Status* |  |  |  |
| Retired | 27.3% (1236) | 10.2% (464) | - |
| Student | 11.0% (498) | 11.9% (540) |  |
| Employed | 40.9% (1853) | 44.1% (2000) |  |
| Freelance | 7.9% (358) | 6.8% (307) |  |
| Unemployed | 6.3% (284) | 6.7% (304) |  |
| Missing Response | 1.0% (46) | 1.4% (64) |  |

⤉ The Swiss equivalent of the education levels listed are scolarité obligatoire, école secondaire, apprentissage, haute école ou université, and doctorat.

* Multiple responses per person allowed for reporting employment status, therefore did not compute global p-value.

**Supplementary Table 6**. Sensitivity analyses using expanded population and a different definition of symptomatic infection. In the first sensitivity analyses (results shown in the 2nd and 3rd columns), in addition to the 2,267 households included in the main analyses, the study population also included 141 households that are only missing blood samples from household members who are 0-4 years old. In the second sensitivity analyses (results shown in the 4th and 5th columns), the study population included all 8,344 enrolled individuals. In the third sensitivity analyses (results shown in the 6th and 7th columns), we considered seropositive individuals who reported symptom onset within the 2 weeks prior to testing asymptomatic. We ran the main model (model 4) for all three sensitivity analyses. Results of the main model presented in supplemental table 2 are also shown here in the 8th and 9th columns for comparison. The main model incorporates individual-level factors including age, sex, and symptom status of potential infectors.

|  | Model 4 sensitivity analyses, household only missing serostatus from 0-4 year olds | | Model 4 sensitivity analyses, all enrolled individuals | | Model 4 sensitivity analyses, alternative definition of symptomatic infection | | Model 4 main analyses | |
| --- | --- | --- | --- | --- | --- | --- | --- | --- |
|  | age + sex + infectors’ symptom + reduced contact *+* extra HH contacts | | age + sex + infectors’ symptom + reduced contact *+* extra HH contacts | | age + sex + infectors’ symptom + reduced contact *+* extra HH contacts | | age + sex + infectors’ symptom + reduced contact + extra HH contacts | |
|  | extra-household | household | extra-household | household | extra-household | household | extra-household | household |
| Category | Odds Ratio (95% CrI) | Odds Ratio (95% CrI) | Odds Ratio (95% rCrI) | Odds Ratio (95% CrI) | Odds Ratio (95% rCrI) | Odds Ratio (95% CrI) | Odds Ratio (95% CrI) | Odds Ratio (95% CrI) |
| Age |  |  |  |  |  |  |  |  |
| 5-9 | 0.4  (0.2, 0.8) | 0.4  (0.1, 1.4) | 0.3  (0.1, 0.6) | 0.3  (0.1, 1.0) | 0.4  (0.2, 1.0) | 0.4 (0.1,1.4) | 0.5 (0.2, 0.9) | 0.4 (0.1, 1.6) |
| 10-19 | 0.5  (0.3, 0.9) | 1.1  (0.4, 2.6) | 0.6  (0.3, 0.8) | 1.3  (0.7, 2.4) | 0.5  (0.2, 0.8) | 0.9  (0.3, 2.3) | 0.5 (0.2, 0.8) | 1.0 (0.4, 2.3) |
| 20-49 | ref | ref | ref | ref | ref | ref | ref | ref |
| 50-64 | 0.7  (0.5, 0.9) | 1.2  (0.5, 2.7) | 0.7  (0.6, 0.9) | 1.2  (0.6, 2.0) | 0.7  (0.5, 1.0) | 1.1  (0.5, 2.3) | 0.7 (0.5, 0.9) | 1.1 (0.5, 2.3) |
| 65+ | 0.4  (0.3, 0.6) | 3.1  (1.0, 8.4) | 0.5  (0.3, 0.6) | 2.8  (1.2, 5.9) | 0.4  (0.3, 0.6) | 2.8  (0.9, 7.9) | 0.4 (0.3, 0.6) | 2.7 (0.9, 7.9) |
| Sex |  |  |  |  |  |  |  |  |
| Male | 1.4  (1.0, 1.8) | 1.2  (0.5, 2.9) | 1.3  (1.0, 1.6) | 1.1  (0.6, 1.9) | 1.4  (1.0, 1.9) | 1.3  (0.6, 2.9) | 1.4 (1.0, 2.0) | 1.4 (0.6, 3.1) |
| Female | ref | ref | ref | ref | ref | ref | ref | ref |
| Symptomatic (infector) |  |  |  |  |  |  |  |  |
| yes | - | 3.5  (1.6, 9.2) | - | 3.1  (1.6, 6.8) | - | 4.1  (1.8, 11.2) | - | 3.3 (1.5, 8.9) |
| no |  | ref |  | ref |  | ref |  | ref |

**Supplementary Table 7**. The number and proportion of households infected

|  | Number rypeople infected in a household (%) | | | | | |
| --- | --- | --- | --- | --- | --- | --- |
| Household size (N) | 0 | 1 | 2 | 3 | 4 | 5 |
| 1 (860) | 819 (95.2) | 41 (4.8) | - | - | - | - |
| 2 (889) | 807 (90.8) | 52 (5.8) | 30 (3.4) | - | - | - |
| 3 (229) | 190 (83.0) | 29 (12.7) | 8 (3.5) | 2 (0.87) | - | - |
| 4 (239) | 188 (78.7) | 38 (15.9) | 4 (1.7) | 5 (2.1) | 4 (2.1) | - |
| 5 (48) | 39 (81.3) | 5 (10) | 2 (4.2) | 1 (2.1) | - | 1 (2.1) |
| 6 (1) | 1 (100) | - | - | - | - | - |
| 7 (1) | 1 (100) | - | - | - | - | - |

**Supplementary Note 1: Technical summary**

1. **Chain binomial model description and main assumptions**

We built a series of models to estimate two quantities: 1) infection risk from extra-household sources and 2) infection risk from a single infected household member. These models are based on an adapted version of chain binomial models ^1,2^ that we fit to the final size of infections within households.

The model assumes that 1) each household member can be infected either from within a household or from extra-household sources, 2) household members mix at random within a household and can infect one another, and 3) all household members were initially susceptible to infection to SARS-CoV-2, and that infection to SARS-CoV-2 confers immunity to reinfection for the duration of the study period. The explicit modeling of chains of transmission accounts for competing risks between community and household infection. In addition, we assume our serological survey fully captures all infections in a household with no misclassification.

We consider all possible sequences of viral introductions to each household and subsequent transmission events within the household. For example, in a household with 2 seropositive individuals, both could have been infected outside of the household, or one could have been infected outside and then infected one other person within the household. For each possible sequence of viral introduction and subsequent transmission events within the household, we assign generation of infections for each household member (i.e., generation $g_{i}$ for household member *i*). So, people infected from outside the household are assigned to generation 0, those they infect to generation 1, those generation 1 infects to generation 2, and so on. Uninfected individuals are assigned an implicit generation of infinity. We augment the data and denote each assignment for all members for household *h* as ${HH}_{h,k}$, where *k* denotes one possible sequence of viral introduction and subsequent transmission events within the household.

We define the probability of a household member *i* escaping infection from a single infectious household member *j* to be $Q_{i,j}$ and the probability of individual *i* escaping infection from the community (i.e., outside household contacts) over the course of epidemic to be $B_{i}$. We define the probability of household member *i* having an infection generation of $g_{i}$as:

$$\Pr\left( g_{i} | \mathrm{HH}_{h,k} \right)=\left( 1-B_{i} \right)^{I\left( g_{i}=0 \right)}\left( B_{i} \right)^{I\left( g_{i}\neq0 \right)}[\prod_{j\neq i,g_{j}<\left( g_{i}-1 \right)} Q_{i,j}][1-\prod_{j\neq i,g_{j}=(g_{i}-1)} Q_{i,j}]$$

where the first three terms,

$$\left( 1-B_{i} \right)^{I\left( g_{i}=0 \right)}\left( B_{i} \right)^{I\left( g_{i}\neq0 \right)}[\prod_{j\neq i,g_{j}<\left( g_{i}-1 \right)} Q_{i,j}]$$

represent the probability of household member *i* escaping infection from extra-household sources and other infected household members up to generation $g_{i}$, and

$$I:=\left\{ \begin{aligned} 1, g_{i}=0 \\ 0, g_{i}\neq0 \end{aligned} \right.$$

is an indicator function that equals to one if household member *i* is infected outside.

$$1-\prod_{j\neq i,g_{j}=g_{i}-1} Q_{i,j}$$

denotes the probability of household member *i* being infected by any infected household member in generation $g_{i}$.

We estimate $Q_{i,j}$ as a function of an exposed individual’s characteristics $\boldsymbol{X}_{\boldsymbol{i}}$ (i.e., age and sex) and the potential infectors’ characteristics $\boldsymbol{X}_{\boldsymbol{j}}$ (i.e, symptoms and/or age).

$$logit\left( Q_{i,j} \right)=\beta_{0}+\boldsymbol{X}_{\boldsymbol{i}}\boldsymbol{\beta}+\boldsymbol{X}_{\boldsymbol{j}}\boldsymbol{\alpha}$$

Similarly, we estimate $B_{i}$, the probability of household member i escaping infection from extra-household sources since the start of the epidemic, as a function of an exposed individual’s

characteristics $\boldsymbol{X}_{\boldsymbol{i}}$ (age and sex) in addition to two variables capturing self-reported behviours related to social contacts: the reduction in social interactions since the start of the pandemic, and the frequency of extra-household social contacts during the pandemic.

$$logit\left( B_{i} \right)=\beta_{0}+\boldsymbol{X}_{\boldsymbol{i}}\boldsymbol{\beta}$$

1. **Inference**

Posterior distributions of parameters are estimated via MCMC using the rstan package, where the likelihood of each observed household pattern of infections is calculated as follows.

The likelihood of each generation assignment is:

$$\Pr\left( {HH}_{h,k} \right)=\prod_{i} \Pr(g_{i}|{HH}_{h,k})$$

And the likelihood of observing the final infection state (i.e., household attack rate) of a household *h* is then:

$$\Pr({HH}_{h,k})=\sum_{k} \Pr({HH}_{h,k})$$

We set weakly informative priors on all parameters to be normally distributed on the logit scale with mean of 0 and standard error of 1.5. We ran four chains of 1,000 iterations each with 250 warm-up iterations and assessed convergence visually and using the Gelman-Rubin Convergence Statistic (R-hat).

1. **Simulation of Infectors**

We simulate the source of infection for all individuals in the study. We first draw one sequence of viral introductions and subsequent within-household transmission events (${HH}_{h,k}$) from all possible sequences for each household, *h*, with at least one seropositive individual. Sequences are drawn for each household with probability vector $\Pr({HH}_{h,k})$ following a categorical distribution,

Next, for each individual, *i*, infected by a household member, we draw the person’s infector from all household members infected in the previous generation. Infectors are drawn from categorical distribution with the probability of each potential infector, *j*, being $1 - Q_{i,j}$[.](https://www.codecogs.com/eqnedit.php?latex=1-Q_%7Bi%2Cj%7D#0)

1. **Handling of missing variables**

As extra-household contact questions were only asked to those over 14 years old, we compared extra-household transmission by self-reported reduction or frequency in social contacts only among those 20 years and older. We imputed a small number of missing response (1%) related to extra-household contacts for those 20 years and older based on responses from other household members. To impute the number of extra-household contacts, we took the midpoint of each category (e.g., 4 if answering 3 to 5 contacts per week) and calculated the average response of other household members. We imputed missing behavior pre- and post-pandemic with the more common response of other household members. Imputing missing data with the average response of those in the same age and sex category as those missing these two variables did not qualitatively change our estimates.

**Supplementary Table 8. Definition of variables and notations**

| Variables | Definition | Notes |
| --- | --- | --- |
| *h* | Household |  |
| *k* | One possible sequence of viral introduction and subsequent transmission events within the household. | In a household of two where both are seropositive, three possible sequences (k=1,2,3) exist: both could have been infected outside of the household, one could have been infected outside and then infected one other person within the household. |
| *i* | Susceptible household member |  |
| *j* | Infectious household member |  |
| *g* | Generation of infection | 0 if infected outside, 1,2,...if infected by another household member |
| $Q_{i,j}$ | The probability of a household member i escaping infection from a single infectious household member j |  |
| $B_{i}$ | The probability of individual i escaping infection from the community (i.e., outside the household) over the course of epidemic |  |
| ${HH}_{h,k}$ | Assignments for all members of household h based on one possible generation sequence k | In a household of two where both are seropositive, ${HH}_{h,1}$= {0,0} if both are infected outside, ${HH}_{h,2}$= {1,0}, and ${HH}_{h,3}$= {0,1} if one is infected outside and subsequently infects the other household member. |
| $X_{i}$ | An exposed individual’s characteristics (e.g., age, sex, and contact variable) |  |
| $X_{j}$ | A potential infector’s characteristics (e.g., age, sex, symptom status) |  |

**Supplementary Table 9. Types of individual characteristics that each model adjusted for for estimating the within household and extra-household transmission risk.**

| Model | Exposed individuals’ characteristics |  | Potential infector’s characteristics |  |
| --- | --- | --- | --- | --- |
|  | extra-household | household | extra-household | household |
| 1 | age | age | - | - |
| 2 | age, sex | age, sex | - | - |
| 3 | age, sex, age/sex interaction | age, sex, age/sex interaction | - | - |
| 4 | age, sex | age, sex | - | symptom |
| 5 | age, sex, reduced-contact | age, sex | - | symptom |
| 6 | age, sex, reduced-contact, extra-household contact | age, sex | - | symptom |
| 7 | age, sex | age, sex | - | symptom, age |
| 8 | sex | sex | - | symptom, age |
| 9 | age, sex | age, sex | - | age |

**Supplementary References**

1. [Fraser, C., Cummings, D. A. T., Klinkenberg, D., Burke, D. S. & Ferguson, N. M. Influenza Transmission in Households During the 1918 Pandemic. *Am. J. Epidemiol.* **174**, 505–514 (2011).](http://paperpile.com/b/IcJpKp/SFYsW)

2. [Longini, I. M., Jr & Koopman, J. S. Household and community transmission parameters from final distributions of infections in households. *Biometrics* **38**, 115–126 (1982).](http://paperpile.com/b/IcJpKp/lWdTD)
