# Supplementary material for: Insights into household transmission of SARS-CoV-2 from a population-based serological survey
Source: Nat Commun. 2021 Jun 15;12:3643. doi: 10.1038/s41467-021-23733-5 (PMC8206123; doi:10.1038/s41467-021-23733-5)
Supplement: Supplementary file 3 — Reporting Summary [file 41467_2021_23733_MOESM3_ESM.pdf]

nature research

Corresponding author(s): NCOMMS-21-05667

Last updated by author(s): May 3, 2021

# Reporting Summary

Nature Research wishes to improve the reproducibility of the work that we publish. This form provides structure for consistency and transparency in reporting. For further information on Nature Research policies, see our Editorial Policies and the Editorial Policy Checklist.

## Statistics

For all statistical analyses, confirm that the following items are present in the figure legend, table legend, main text, or Methods section.

| n/a | Confirmed | |
|---|---|---|
| ☐ | ☒ | The exact sample size (*n*) for each experimental group/condition, given as a discrete number and unit of measurement |
| ☐ | ☒ | A statement on whether measurements were taken from distinct samples or whether the same sample was measured repeatedly |
| ☐ | ☒ | The statistical test(s) used AND whether they are one- or two-sided<br>*Only common tests should be described solely by name; describe more complex techniques in the Methods section.* |
| ☐ | ☒ | A description of all covariates tested |
| ☒ | ☐ | A description of any assumptions or corrections, such as tests of normality and adjustment for multiple comparisons |
| ☐ | ☒ | A full description of the statistical parameters including central tendency (e.g. means) or other basic estimates (e.g. regression coefficient) AND variation (e.g. standard deviation) or associated estimates of uncertainty (e.g. confidence intervals) |
| ☐ | ☒ | For null hypothesis testing, the test statistic (e.g. *F*, *t*, *r*) with confidence intervals, effect sizes, degrees of freedom and *P* value noted<br>*Give P values as exact values whenever suitable.* |
| ☐ | ☒ | For Bayesian analysis, information on the choice of priors and Markov chain Monte Carlo settings |
| ☒ | ☐ | For hierarchical and complex designs, identification of the appropriate level for tests and full reporting of outcomes |
| ☐ | ☒ | Estimates of effect sizes (e.g. Cohen's *d*, Pearson's *r*), indicating how they were calculated |

*Our web collection on statistics for biologists contains articles on many of the points above.*

## Software and code

Policy information about availability of computer code

| | |
|---|---|
| Data collection | None |
| Data analysis | Code needed to reproduce results are available at https://github.com/HopkinsIDD/serocovpop-households. We used rstan package (version 2.21.0) in R (version 3.6.3) for primary analyses |

For manuscripts utilizing custom algorithms or software that are central to the research but not yet described in published literature, software must be made available to editors and reviewers. We strongly encourage code deposition in a community repository (e.g. GitHub). See the Nature Research guidelines for submitting code & software for further information.

## Data

Policy information about availability of data

All manuscripts must include a data availability statement. This statement should provide the following information, where applicable:

- Accession codes, unique identifiers, or web links for publicly available datasets
- A list of figures that have associated raw data
- A description of any restrictions on data availability

Data can be made available to share upon submission of a data request application to the investigators board via the corresponding author or Dr. Silvia Stringhini (silvia.stringhini@hcuge.ch).

# Field-specific reporting

Please select the one below that is the best fit for your research. If you are not sure, read the appropriate sections before making your selection.

- [x] Life sciences
- [ ] Behavioural & social sciences
- [ ] Ecological, evolutionary & environmental sciences

For a reference copy of the document with all sections, see nature.com/documents/nr-reporting-summary-flat.pdf

# Life sciences study design

All studies must disclose on these points even when the disclosure is negative.

| | |
|---|---|
| Sample size | Secondary analysis or original study so sample size determined by original study enrollment. |
| Data exclusions | Primary analyses were based on households with complete enrollment, thus we excluded households where not all members provided blood samples. This exclusion criteria was pre-established, because our statistical methods require fitting models to data from households with complete serostatus. We did not have sufficient data on missing individuals from incomplete household to use imputation procedures. |
| Replication | Code for reproducing results of the statistical analyses were available at https://github.com/HopkinsIDD/serocovpop-households. |
| Randomization | Not relevant to this study. Study participants were not allocated to experimental groups. |
| Blinding | Not relevant to this study. Study participants were not allocated to experimental groups, thus binding to group allocation was not necessary. |

# Reporting for specific materials, systems and methods

We require information from authors about some types of materials, experimental systems and methods used in many studies. Here, indicate whether each material, system or method listed is relevant to your study. If you are not sure if a list item applies to your research, read the appropriate section before selecting a response.

## Materials & experimental systems

| n/a | Involved in the study | |
|---|---|---|
| x | | Antibodies |
| x | | Eukaryotic cell lines |
| x | | Palaeontology and archaeology |
| x | | Animals and other organisms |
| | x | Human research participants |
| x | | Clinical data |
| x | | Dual use research of concern |

## Methods

| n/a | Involved in the study | |
|---|---|---|
| x | | ChIP-seq |
| x | | Flow cytometry |
| x | | MRI-based neuroimaging |

## Human research participants

Policy information about studies involving human research participants

| | |
|---|---|
| Population characteristics | Individuals randomly selected from the general population of Geneva canton in the SeroCov-POP study aged 5 years and above. About half were female (53.6%) and the median age of participants was 53 years old. Over 6.6% of individuals tested positive for SARS-CoV-2 anti-S1 IgG antibodies. |
| Recruitment | The SEROCoV-POP is a cross-sectional population-based survey of former participants of an annual survey of individuals 20-74 years old representative of the population of Geneva (Canton), Switzerland. The enrollment into the study occurred from April through June 2020 during the first wave of the SARS-CoV-2 pandemic in Geneva. First wave lockdown measures (including school closures) started in mid-March and largely ended by the end of May. The full survey protocols is available online and a detailed description of the design and seroprevalence results were previously published.<br><br>The SEROCoV-POP study invited all 10,587 participants of the previous annual surveys to participate in the through email or post. Participants were invited to bring all members of their household aged 5 years and older to join the study. After providing informed written consent, participants either filled out a questionnaire online, in the days before their visit, or at the time of their visit at one of two enrollment locations (the main canton hospital and one satellite location) within Geneva.<br><br>Risk of extra-household and intra-household transmission could differ as household composition of enrolled study participants change and if behavioral pattern of our study participants are not representative of the pattern in the general canton population. However, relative risk by age and symptom status within households should be not be largely affected by the representativeness of our study population. |
| Ethics oversight | The study was approved by the Cantonal Research Ethics Commission of Geneva, Switzerland (CER16-363) |

Note that full information on the approval of the study protocol must also be provided in the manuscript.
